# Supplementary material for: Comprehensive Annotation and Functional Exploration of MicroRNAs in Lettuce
Source: Front Plant Sci. 2021 Dec 24;12:781836. doi: 10.3389/fpls.2021.781836 (PMC8739914; doi:10.3389/fpls.2021.781836)
Supplement: Supplementary file 2 [file Data_Sheet_8.ZIP › GSM2230749.plot/Lsa-miRN1664_Lsat_1_v5_gn_8_10841.1_2799_TPlot.pdf]

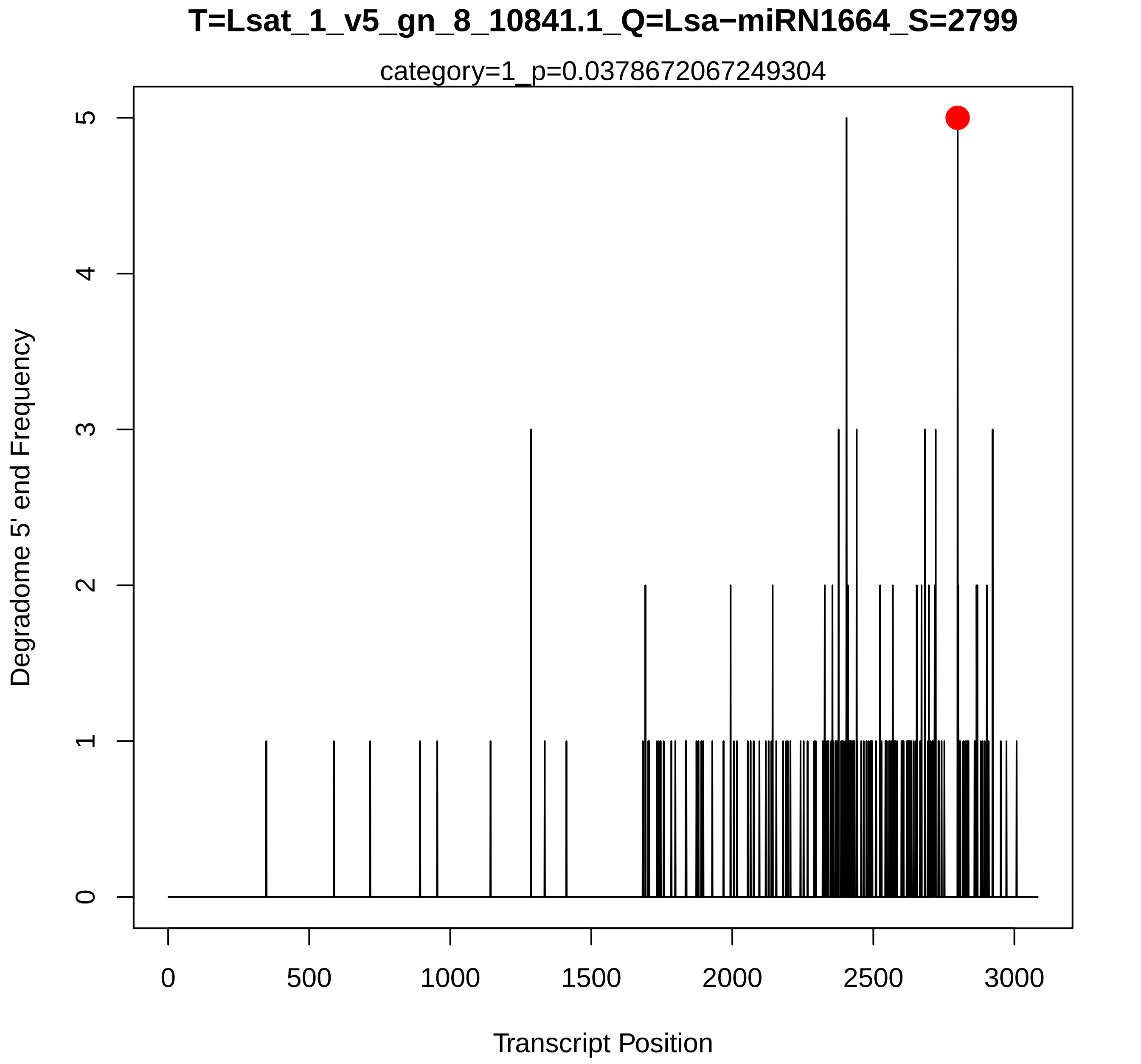

T=Lsat_1_v5_gn_8_10841.1_Q=Lsa−miRN1664_S=2799
category=1_p=0.0378672067249304
Degradome 5' end Frequency
Transcript Position
0
1
2
3
4
5
0
500
1000
1500
2000
2500
3000
